# Management of Patients Diagnosed with Endometrial Hyperplasia: Comparison of Guidelines

**DOI:** 10.3390/cancers18132048

**Published:** 2026-06-24

**Authors:** Stefano Restaino, Chiara Paglietti, Federico Paparcura, Bogani Giorgio, Capozzi Vito Andrea, Ursula Catena, Antonio Raffone, Maria Orsaria, Carlo Ronsini, Giuseppe Scibilia, Tommaso Simoncini, Diego Raimondo, Violante Di Donato, Muhammed Elhadi, Giampiero Capobianco, Ramon Rovira Negre, Anna Biasioli, Monica Della Martina, Mariuzzi Laura, Stefano Uccella, Andrea Ciavattini, Errico Zupi, Renato Seracchioli, Lorenza Driul, Paolo Scollo, Anna Miryam Perrone, Pierandrea De Iaco, Martina Arcieri, Francesco Fanfani, Giuseppe Vizzielli, on behalf of the Collaborative Group

**Affiliations:** 1 Clinic of Obstetrics and Gynecology, “Santa Maria della Misericordia” University Hospital, Azienda Sanitaria Universitaria Friuli Centrale, 33100 Udine, Italy; stefano.restaino@asufc.sanita.fvg.it (S.R.); chiara.paglietti@asufc.sanita.fvg.it (C.P.); anna.biasioli@asufc.sanita.fvg.it (A.B.); monica.dellamartina@asufc.sanita.fvg.it (M.D.M.); lorenza.driul@uniud.it (L.D.); giuseppevizzielli@yahoo.it (G.V.); 2 PhD School in Biomedical Sciences, Gender Medicine, Child and Women Health, University of Sassari, 07100 Sassari, Italy; 3 Department of Medicine, University of Udine, 33100 Udine, Italy; 4 Gynecological Oncology Unit, Fondazione IRCCS Istituto Nazionale dei Tumori, 20133 Milan, Italy; giorgio.bogani@istitutotumori.mi.it; 5 Department of Medicine and Surgery, University Hospital of Parma, 43126 Parma, Italy; vitoandrea.capozzi@studenti.pr.it; 6 Department of Woman, Child and Public Health, Division of Gynecologic Oncology, Fondazione Policlinico Universitario A. Gemelli-IRCCS, 00168 Rome, Italy; ursula.catena@policlinicogemelli.it (U.C.);; 7 Department of Woman, Child, and General and Specialized Surgery, University of Campania “Luigi Vanvitelli”, 80138 Naples, Italy; antonio.raffone@unicampania.it; 8 Institute of Pathology, “Santa Maria della Misericordia” University Hospital, Azienda Sanitaria Universitaria Friuli Centrale, 33100 Udine, Italy; maria.orsaria@asufc.sanita.fvg.it (M.O.); laura.mariuzzi@asufc.sanita.fvg.it (M.L.); 9 Unit of Gynecologic Oncology, National Cancer Institute, IRCCS, Fondazione “G. Pascale”, 80131 Naples, Italy; carlo.ronsini@unicampania.it; 10 Obstetrics and Gynecology, “Giovanni Paolo II” Hospital, 97100 Ragusa, Italy; g.scibilia@libero.it; 11 Department of Clinical and Experimental Medicine, University of Pisa, 56126 Pisa, Italy; tommaso.simoncini@med.unipi.it; 12 Division of Gynecology and Human Reproduction Physiopathology, IRCCS Azienda Ospedaliero-Universitaria di Bologna, 40138 Bologna, Italy; diego.raimondo2@unibo.it (D.R.); renato.seracchioli@unibo.it (R.S.); 13 Department of Maternal and Child Health and Urological Sciences, Policlinico Umberto I, Sapienza University of Rome, 00185 Rome, Italy; violante.didonato@uniroma1.it; 14 College of Medicine (Elhadi), Korea University, Seoul 02841, Republic of Korea; muhammed-elhadi@korea.ac.kr; 15 Department of Medicine, Surgery and Pharmacy, University of Sassari, 07100 Sassari, Italy; capobia@uniss.it; 16 Department of Gynecology and Obstetrics, Hospital de la Santa Creu i de Sant Pau, C/Sant Quintí 89, 08041 Barcelona, Spain; rroviran@santpau.cat; 17 Unit of Obstetrics and Gynecology, Department of Surgery, Dentistry, Pediatrics, and Gynecology, AOUI, 37134 Verona, Italy; stefano.uccella@univr.it; 18 Gynecologic Section, Department of Odontostomatologic and Specialized Clinical Science, Università Politecnica delle Marche, 60123 Ancona, Italy; a.ciavattini@staff.univpm.it; 19 Department of Molecular and Developmental Medicine, Obstetrics and Gynecological Clinic, University of Siena, 53100 Siena, Italy; errico.zupi@unisi.it; 20 Maternal and Child Department, Obstetrics and Gynecology, Cannizzaro Hospital, 95100 Catania, Italy; paolo.scollo@unikore.it; 21 Division of Oncologic Gynecology, IRCCS Azienda Ospedaliero-Universitaria di Bologna, Via Massarenti 9, 40138 Bologna, Italy

**Keywords:** hyperplasia, endometrial, management, fertility, progestogen, cancer

## Abstract

Endometrial hyperplasia (EH) is an estrogen-driven proliferative disorder with a measurable risk of progression to endometrial carcinoma. Although many guidelines have been issued over the years, clinical practice remains heterogeneous. In this paper, we aim to compare and summarize key recommendations and disagreements among major international guidelines for managing endometrial hyperplasia, focusing especially on conservative and fertility-sparing strategies. All guidelines align with some key principles: they all adopt the 2020 WHO classification, strongly prefer hysteroscopy-directed sampling, and recommend progestin therapy as the first-line treatment for non-atypical EH, favoring the levonorgestrel-releasing intrauterine system (LNG-IUS) over oral regimens. They designate total hysterectomy as definitive management for atypical hyperplasia/intraepithelial endometrial neoplasia (AEH/EIN) due to the substantial prevalence of concurrent carcinoma. Nevertheless, several key discrepancies appear, mainly concerning how long to continue progestin therapy and when to escalate treatment; and how intensively and for how long to conduct post-treatment surveillance. Variations in diagnostic and therapeutic protocols reflect evidence gaps and differences across healthcare settings. Future research should focus on harmonized outcomes, comparative studies of conservative strategies, and the integration of new pathology tools for personalized management.

## Simple Summary

Endometrial hyperplasia has a high epidemiological impact, and its management is part of everyday practice. This review is proposed to address persistent inconsistencies in the diagnosis, treatment, surveillance, and fertility-sparing management of endometrial hyperplasia across international guidelines. The authors aim to compare and synthesize recommendations from major gynaecologic societies, identify areas of agreement and disagreement, and provide a practical evidence-based management approach. By highlighting current evidence gaps and variations in clinical practice, the study may support the development of more standardized, personalized, and globally harmonized care pathways.

## 1. Introduction

Endometrial hyperplasia is an estrogen-dependent pathological condition characterized by a wide spectrum of morphological alterations, ranging from benign changes to lesions with neoplastic potential [1]. Histologically, it is defined as an irregular proliferation of endometrial glands, resulting in an increased gland-to-stroma ratio compared with the normal endometrium [2]. In most cases, endometrial hyperplasia develops in the context of estrogen excess. Estrogens, whether endogenous or exogenous, physiologically stimulate endometrial proliferation; thus, all conditions that lead to unopposed estrogen exposure represent a possible risk factor. These include obesity, unbalanced estrogen therapy, tamoxifen use, and chronic anovulation (as seen in perimenopause, polycystic ovary syndrome, or infertility) [3,4,5,6]. Other contributing factors are estrogen-secreting tumors, early menarche, and late menopause [7]. Moreover, women with Lynch syndrome have a markedly higher risk of atypical hyperplasia and endometrial carcinoma [8,9,10]. Over time, several histopathologic classification systems have been developed to better stratify endometrial hyperplasia according to its biological behavior and risk of malignant progression [11,12,13]. Although international societies endorse diagnostic terminology that clearly distinguishes entities requiring distinct management pathways, debate persists over which system offers greater accuracy and practicality. Given persistent limitations of current classifications, including variability in interpretation and incomplete integration of molecular predictors, there is growing recognition that refined or combined stratification models may further improve risk assessment and guide personalized management strategies. The most common sign of endometrial hyperplasia is unusual bleeding from the uterus. Heavy periods, bleeding between periods, and postmenopausal bleeding often lead women to see a gynecologist, who can help diagnose the condition early. Sometimes, it is found by chance in women without symptoms during a regular ultrasound or imaging for other reasons. In these cases, it may be suspected that abnormal cells are seen during cervical screening. Sometimes, hyperplasia or cancer is found unexpectedly in a uterus removed for unrelated problems. It is very important to find endometrial hyperplasia early. Without treatment, especially for the atypical type, it can lead to cancer of the uterus lining. Over the years, international societies have developed guidelines to minimize variations in clinical practice and to improve patient outcomes. The purpose of this paper is to summarize and compare the similarities and major differences among current clinical guidelines for the management of endometrial hyperplasia. Few studies have specifically compared international guidelines for the management of endometrial hyperplasia. A recent review by Boureka et al. provided a comparison of recommendations from ACOG, RCOG, and SOGC. The present review expands upon this analysis by including additional national and international guidelines, allowing a broader assessment of areas of consensus and disagreement and providing a more global perspective on the management of endometrial hyperplasia. By clarifying these commonalities and disparities, the paper aims to assist clinicians in decision-making and optimize patient care.

## 2. Materials and Methods

This is a descriptive comparative review. A literature search was conducted in PubMed and on the official websites of national and international gynaecologic societies to identify guidelines and consensus documents addressing the diagnosis, treatment, and follow-up of endometrial hyperplasia published between January 2000 and December 2025. Guidelines were eligible for inclusion if they provided specific recommendations on the management of endometrial hyperplasia and represented the most recent complete version issued by the respective scientific society. Superseded versions, duplicate publications, and documents not specifically focused on endometrial hyperplasia were excluded. The retrieved documents included the American College of Obstetricians and Gynecologists (ACOG, 2023) Clinical Consensus [14], the Royal College of Obstetricians and Gynecologists (RCOG, 2016) Green-top Guideline [15], the Society of Obstetricians and Gynecologists of Canada (SOGC, 2019) Guideline [16], and the Chinese guideline on the management of endometrial hyperplasia (2024) [17]. A particular focus is given to the fertility-sparing management of patients with endometrial hyperplasia, as women tend to pursue pregnancy at older ages, and the condition is being diagnosed in younger women. For this reason, we also included the Italian Guidelines from the GISS Gynaecologic Oncology Working group (2020) [18] and the ESGO/ESHRE/ESGE Guidelines for the fertility-sparing treatment of patients with endometrial carcinoma (2023) [19], which incorporate the management of patients with atypical hyperplasia. For each society, the most recent complete version of the guideline available at the time of manuscript preparation was selected. Recommendations were extracted from the full-text guidelines and grouped into predefined clinical domains, including diagnostic assessment, treatment of endometrial hyperplasia without atypia, treatment of atypical hyperplasia/endometrial intraepithelial neoplasia (EIN), fertility-sparing management, and follow-up. The recommendations were then summarized in comparative tables and analyzed descriptively to identify areas of concordance and divergence among the included guidelines. As this was a descriptive-comparative review, no formal ranking or quality appraisal of the included guidelines was performed. Guidelines were selected based on their relevance to the management of endometrial hyperplasia and the availability of the most recent complete version published by the respective scientific society. Recommendations were extracted and compared as reported in the original documents. When differences or conflicting recommendations were identified, these were presented and discussed descriptively to highlight areas of agreement, divergence, and uncertainty in current clinical practice, rather than being adjudicated by the authors. A summary of the recommendations is provided in Table 1 (diagnostic assessment), Table 2 (endometrial hyperplasia without atypia), and Table 3 (atypical hyperplasia/endometrial intraepithelial neoplasia). The corresponding levels of evidence are reported when available.

## 3. Results

To facilitate a clear and comprehensive comparison across guidelines, we structured our results into thematic sections by key domains of current clinical interest, thereby enabling a focused and systematic synthesis of convergences and discrepancies.

### 3.1. Terminology/Classification

Over the years, three main classification systems have been developed to categorize endometrial hyperplasia based on histopathological features and the likelihood of progression to malignancy.

The 1994 WHO Classification identified four distinct entities according to glandular architectural complexity and the presence of nuclear atypia: simple hyperplasia without atypia (1% risk of progression to carcinoma), complex hyperplasia without atypia (3%), simple atypical hyperplasia (8%), and complex atypical hyperplasia (29%). This descriptive approach was limited by poor inter-observer reproducibility and inadequate guidance for appropriate clinical management, often leading to overtreatment [11]. The 2014 WHO Classification simplified the system into two categories, independent of architectural complexity:

Endometrial hyperplasia without atypia (benign form), characterized by pseudostratified columnar cells and irregular, dilated glands.

Atypical endometrial hyperplasia/endometrial intraepithelial neoplasia (EIN), showing cytological and architectural atypia with crowded, irregular glands lacking intervening stroma [2].

The classification was revised in 2020, maintaining the distinction between endometrial hyperplasia without atypia and endometrial intraepithelial neoplasia (EIN)/atypical hyperplasia. Although no major changes in terminology were introduced for these lesions, the updated classification reinforced the concept that EIN and atypical hyperplasia represent equivalent premalignant conditions associated with a substantial risk of progression to endometrial carcinoma [12].

In 2000, the International Endometrial Collaborative Group proposed the EIN system, integrating histological, molecular, and morphometric parameters, including the D-score (ratio of stromal volume to total tissue volume). This approach aimed to enhance diagnostic precision and prognostic accuracy while minimizing observer variability [13]. The EIN system distinguishes three categories:

Benign endometrial hyperplasia, ranging from proliferative-type endometrium to cystic glandular hyperplasia.

Endometrial intraepithelial neoplasia (EIN) is a premalignant lesion with crowded glands and cytological atypia.

Well-differentiated endometrioid adenocarcinoma, showing stromal invasion.

While the EIN system offers greater sensitivity, especially when incorporating objective measures like the D-score. It is not always feasible in routine practice due to technical and economic limitations. Recent comparative studies between WHO and EIN (without D-score) classifications suggest overall prognostic equivalence. However, EIN demonstrates higher sensitivity in identifying women who require treatment, whereas the WHO system, which relies primarily on cytologic atypia, is more specific in recognizing lesions at greatest risk of malignant transformation [20,21]. The optimal classification system for diagnosis and management remains a matter of debate. All guidelines included in this review adhere to the revised 2020 World Health Organization classification of endometrial hyperplasia.

### 3.2. Diagnostic and Surveillance Methods

Endometrial hyperplasia should be suspected in women presenting with abnormal uterine bleeding. Transvaginal ultrasound serves as an initial diagnostic tool to assess endometrial morphology and thickness; however, the definitive diagnosis is histopathological, requiring endometrial tissue sampling. Biopsy is also essential for monitoring disease regression, persistence, or progression. A thorough clinical history and physical examination are crucial. The anamnesis should include evaluation of comorbidities, prior surgeries, use of medications or hormone replacement therapy (HRT)/tamoxifen, menstrual characteristics, peri- or postmenopausal status, obstetric history, fertility intentions, and the nature of bleeding symptoms. The physical examination should comprise both a general assessment recording vital signs, particularly in women with heavy bleeding or anemia, and a detailed abdominal and gynaecologic examination, including inspection of external genitalia, speculum evaluation of the cervix and vagina, and bimanual palpation to assess uterine size, shape, and adnexal structures. Moreover, it could include a Pap smear/HPV determination for differential diagnosis.

Pelvic ultrasonography is valuable for identifying focal lesions (such as polyps or submucosal fibroids), endometrial heterogeneity, or thickening. RCOG guidelines underline that diagnostic thresholds vary by age and reproductive status.

Recent evidence has refined the interpretation of endometrial thickness measurements and the indications for endometrial sampling in different clinical settings. In premenopausal women, physiological endometrial thickness fluctuates with the menstrual cycle, making it challenging to distinguish normal from pathological endometrium; nonetheless, a cutoff of 7 mm has been suggested, below which hyperplasia is highly unlikely. In postmenopausal women presenting with bleeding, a threshold of ≤4 mm remains associated with a very low likelihood of endometrial cancer, and endometrial biopsy is generally recommended when thickness exceeds 4 mm or when bleeding persists despite a thin endometrium. Conversely, in asymptomatic postmenopausal women, no universally accepted cut-off exists, and routine endometrial biopsy is not recommended solely based on a mildly increased endometrial thickness. Recent evidence-based reviews suggest that a threshold of approximately 10–11 mm may represent a reasonable trigger for further investigation, particularly in the presence of additional risk factors for endometrial carcinoma. Vitale et al. highlighted the lack of consensus regarding the optimal biopsy threshold in asymptomatic women, whereas several contemporary studies and systematic reviews have reported that the risk of endometrial cancer or atypical hyperplasia increases substantially when the thickness reaches or exceeds 11 mm. More recently, Wang et al. proposed a lower threshold of 8 mm for biopsy in asymptomatic postmenopausal women, with individualized assessment for women with endometrial thickness between 4 and 8 mm according to clinical risk factors and ultrasound characteristics [22,23,24,25].

Special consideration is required for women receiving hormone replacement therapy or tamoxifen. In tamoxifen users, routine endometrial surveillance based solely on ultrasound is not recommended because tamoxifen-induced stromal changes may artificially increase sonographic thickness; diagnostic evaluation should instead be driven primarily by symptoms, especially postmenopausal bleeding. Although an endometrial thickness threshold of >8 mm has traditionally been proposed for further investigation in women receiving tamoxifen, current evidence does not support routine endometrial evaluation in asymptomatic premenopausal or postmenopausal tamoxifen users. Similarly, in asymptomatic women receiving hormone replacement therapy, a threshold of up to 8 mm is generally considered acceptable in the absence of additional suspicious ultrasonographic findings, and routine endometrial sampling is not recommended solely on the basis of endometrial thickness [24,26].

#### 3.2.1. Endometrial Sampling

Across major international guidelines, histologic sampling is consistently emphasized as essential for diagnosing endometrial hyperplasia. The ACOG recommends hysteroscopic-guided sampling, highlighting its superior ability to diagnose polyps, hyperplasia, and carcinoma and cautioning against techniques that produce crushed or cauterized tissue, which may compromise histologic interpretation. Special attention is given to tissue removal devices that can provide simple endometrial resection, morcellate the specimen in a contained environment, and produce excellent samples for evaluation. The RCOG similarly supports the use of diagnostic hysteroscopy, particularly when an outpatient biopsy is inadequate or when hyperplasia is suspected within a focal lesion, as hysteroscopy allows direct visualization and targeted biopsy. While an outpatient endometrial biopsy is convenient, RCOG notes its lower accuracy for diagnosing hyperplasia and the possibility of missed disease [27,28]. The Chinese guidelines support the use of hysteroscopy as a safe and effective method for assessing the uterine cavity and obtaining targeted endometrial biopsies. Nevertheless, they do not prioritize hysteroscopy over dilation and curettage (D&C), considering both procedures acceptable and equivalent options for diagnostic endometrial sampling. ESGO/ESHRE/ESGE Guidelines recommend avoiding blind techniques and support a shift toward visually directed hysteroscopic sampling. Specifically, the “grasp” biopsy technique is preferred because it allows the collection of larger, more representative endometrial tissue fragments. In cases where the endometrium is atrophic or hypo trophic, adequate sampling can be achieved using bipolar electrodes, fine scissors, or intrauterine tissue removal systems, which allow precise and sufficient retrieval of diagnostic material. In contrast, the SOGC states that all standard sampling methods are generally reliable but cites strong evidence from a large meta-analysis showing that the Pipelle device has the highest sensitivity (91% in premenopausal women, 99.6% in postmenopausal women for detecting carcinoma, and 81% for detecting atypical hyperplasia) [29]. However, these blind sampling approaches typically evaluate less than 50% of the endometrial cavity and may yield insufficient tissue. Therefore, when the biopsy is non-diagnostic, symptoms persist, suspicion remains high, or the procedure is technically limited, diagnostic hysteroscopy with directed sampling and curettage is recommended as the preferred next step. Overall, all the guidelines recognize hysteroscopy as a valuable tool, particularly in cases of nondiagnostic outpatient sampling, focal lesions, or when excluding higher-grade disease prior to treatment.

#### 3.2.2. Imaging

Computed tomography and magnetic resonance imaging play a limited role in the management of endometrial hyperplasia. Both are costly examinations, and there is insufficient evidence in the literature to support their use for the diagnosis or surveillance of women with atypical hyperplasia undergoing conservative management. ACOG, SOGC, and Italian Guidelines do not discuss their use in this setting. RCOG guidelines state that CT and diffusion-weighted MRI are not routinely indicated, noting that while preoperative CT may alter management in a small proportion of cases, there is no evidence supporting their use in follow-up. Diffusion-weighted MRI may detect invasive disease or predict malignant progression, but further validation is required before it can be incorporated into standard care. Chinese guidelines support the use of MRI to detect invasive carcinoma.

### 3.3. Management of Non-Atypical Endometrial Hyperplasia

Effective management of newly diagnosed endometrial hyperplasia requires clear objectives: preventing progression to carcinoma, detecting coexisting cancer, and tailoring treatment to individual characteristics, risk factors, and reproductive goals. The appropriate approach depends on histological severity, menopausal status, and fertility or contraception needs.

In endometrial hyperplasia without atypia, the risk of progression to endometrial carcinoma is low, and therefore, a conservative management approach is appropriate in most cases. Long-term cohort data indicate a reassuring cumulative progression risk of <5% over 20 years [30,31,32]. A more recent cohort study including women initially diagnosed with non-atypical endometrial hyperplasia, progression to AH or cancer was seen in 13% after a median follow-up of 5 years [33]. These findings support an individualized, conservative strategy, particularly after correction of contributing factors, while ensuring ongoing surveillance to detect persistence or progression. Current professional society guidance on endometrial hyperplasia without atypia generally emphasizes a conservative, individualized management approach. The RCOG notes that progression risk is low (<5% over 20 years), with most cases regressing spontaneously, especially when reversible risk factors like obesity or unopposed estrogen exposure are addressed. Observation with scheduled endometrial biopsies may therefore be considered, although women should be informed that progestogen therapy achieves higher regression rates than surveillance alone and is recommended for those with persistent disease or symptomatic bleeding. The SOGC similarly supports beginning with conservative management, given the generally favorable natural course of endometrial hyperplasia without atypia. Conversely, Chinese guidelines do not support conservative management as first-line therapy for hyperplasia without atypia, especially in cases with high risk. This approach reflects concerns regarding the uncertain duration of safe observation and the relatively high likelihood of persistent disease. However, all patients should be counseled on strategies to address modifiable risk factors associated with endometrial hyperplasia and endometrial cancer. In contrast, ACOG, GISS-SIGO, and ESGO–ESHRE do not specifically address risk-factor modification or conservative management strategies in this setting.

#### 3.3.1. Addressing Risk Factors

Because the progression of endometrial hyperplasia without atypia to cancer is typically slow, there is a meaningful opportunity to identify and modify reversible risk factors as part of management. Obesity is one of the strongest contributors to excess endogenous estrogen exposure. While evidence on structured weight-loss strategies specifically improving hyperplasia outcomes is limited, observational studies indicate that up to 10% of severely obese, asymptomatic women may harbor undiagnosed hyperplasia. Bariatric surgery may reduce this risk. Clinicians should also carefully review the use of exogenous estrogens, including prescribed hormone replacement therapy and nonprescription preparations that may contain unregulated estrogenic compounds. Adjusting the estrogen–progestogen balance in hormone therapy alone may be sufficient to induce regression in some postmenopausal women. Additionally, tamoxifen therapy should be reassessed in collaboration with oncology specialists. Finally, addressing anovulation, a common driver of unopposed estrogen exposure in women with PCOS or during the perimenopausal transition, may support spontaneous regression. Resumption of ovulatory cycles can restore normal progesterone-mediated endometrial regulation [34,35,36,37].

#### 3.3.2. Medical Management

Across guidelines, progestin therapy is consistently recommended as the primary medical treatment for endometrial hyperplasia without atypia, with unanimous support for the levonorgestrel-releasing intrauterine system (LNG-IUS) as the preferred first-line option. The RCOG highlights that LNG-IUS achieves higher regression rates, a more favorable bleeding profile, and fewer systemic adverse effects than oral progestins, supporting its use over continuous oral regimens [38,39]. A meta-analysis cited by both RCOG and SOGC confirms higher regression rates with the LNG-IUS compared with oral progestins, with reduced need for hysterectomy [40]. Continuous progestogens should be used for women who decline the LNG-IUS, while cyclical progestogens should not be used because they are less effective in inducing regression. Different progestogen types, doses, and regimens have been proposed, but their efficacy appears comparable [41,42]. The SOGC also notes that injectable medroxyprogesterone, aromatase inhibitors, or letrozole may be effective alternatives in selected premenopausal or postmenopausal patients [43,44]. The Chinese guidelines likewise recommend the LNG-IUS as first-line therapy, with continuous oral progestins as an acceptable alternative when the device is declined or contraindicated. Oral progestin treatment should be used for at least 3–6 months, while LNG-IUS can be used long-term with regular replacement. Alternative options include combined oral contraceptives, aromatase inhibitors, and gonadotropin-releasing hormone agonists. However, it is emphasized that evidence confirming the effectiveness of these drugs is lacking; thus, patients should be informed that these drugs are experimental or being used off-label.

In contrast, ACOG, SIGO, and ESGO–ESHRE do not provide specific recommendations on medical therapy in this context. The various dosage regimens are summarized in Table 2. Both RCOG, SGOC, and Chinese guidelines agree that a minimum treatment duration with oral progestogens or the LNG-IUS should be 6 months to induce histological regression of endometrial hyperplasia without atypia. They all agree that if adverse effects are tolerable and fertility is not desired, women should be encouraged to retain the LNG-IUS for up to 5 years, as this reduces the risk of relapse, especially if it alleviates abnormal uterine bleeding symptoms.

#### 3.3.3. Follow up

All guidelines emphasize the importance of a structured follow-up with endometrial sampling during and after medical treatment, although surveillance intervals and duration vary. Endometrial sampling can be performed with the intrauterine device in place. The RCOG recommends surveillance at a minimum 6-month intervals, individualized according to clinical risk; women with a BMI ≥ 35 or those treated with oral progestins are considered at higher risk of relapse and should undergo annual biopsies after two consecutive negative samples. Longer treatment duration improves outcomes, with regression rates increasing between 3 and 6 months substantially (from 84% to 100% for the LNG-IUS and from 50% to 64% for oral MPA) [45]. The SOGC similarly advises endometrial biopsy every 3–6 months during treatment and recommends reassessing therapy if there is no response after 6 months, with a change in management if no regression is observed by 12 months. Both RCOG and SOGC note higher relapse rates in women with elevated BMI, warranting extended monitoring. According to the Chinese guidelines, patients should undergo transvaginal ultrasonography and endometrial histological assessment at six-month intervals. Hysteroscopic follow-up may be discontinued only after two consecutive endometrial biopsies demonstrate complete histological regression. Similar to other guidelines, if complete remission is not achieved after 6 months of drug treatment, the patient should be informed of other treatment options, and if complete remission is not achieved after 12 months, surgical management should be considered. Upon discharge from follow-up, all guidelines stress the need to educate patients to seek a further referral if abnormal vaginal bleeding recurs, which may indicate disease relapse.

#### 3.3.4. Surgical Management

Surgical treatment should not be considered a first-line treatment for endometrial hyperplasia without atypia, as progestin therapy achieves clinical and histologic regression in most patients, allowing a conservative approach and avoiding the morbidity associated with definitive surgery. However, SOCG affirms that hysterectomy is appropriate in women who do not desire future fertility and who experience progression to atypical hyperplasia or carcinoma during follow-up, whose hyperplasia fails to regress after 12 months of medical treatment or relapses after completing, who continue to experience abnormal uterine bleeding despite treatment, or who decline endometrial surveillance or medical treatment. RCOG and Chinese guidelines recommend hysterectomy if endometrial hyperplasia persists for 12 months despite treatment, as the risk of underlying cancer is high and the chances of disease regression are low. ACOG, SIGO, and ESGO–ESHRE do not provide specific recommendations on surgical therapy in this context. SOCG specifically advises performing a total hysterectomy with salpingectomy, with or without bilateral oophorectomy. Ovarian conservation is recommended in premenopausal women at average risk, as bilateral oophorectomy is associated with increased long-term morbidity and mortality. Opportunistic salpingectomy should be performed to reduce ovarian cancer risk. In postmenopausal women, the decision should be individualized, with a tendency toward oophorectomy in older patients [46,47].

### 3.4. Management of Atypical Endometrial Hyperplasia

Atypical endometrial hyperplasia requires careful assessment due to its high risk of progression to carcinoma. In women diagnosed with this condition, foci of endometrioid adenocarcinoma may already be present, or the lesion may progress over time to malignancy. A literature review including 2572 patients reported that 37% of women with a biopsy diagnosis of atypical endometrial hyperplasia were found to have invasive carcinoma on subsequent biopsy or hysterectomy specimen. Advanced age, obesity, diabetes, and the presence of cytologic atypia are the clinical factors most strongly associated with coexisting invasive adenocarcinoma in women with endometrial hyperplasia. The rate of progression to invasive malignancy is strongly correlated with the persistence of the underlying etiopathogenetic condition over time [48,49]. Several key studies have evaluated progression risk. The largest case–control study found that EH with atypia has a cumulative risk of progression to endometrial cancer that increases from 8.2% in the first four years to 12.4% at nine years, up to 27.5% (95% CI 8.6% to 42.5%) at nineteen years from diagnosis [31]. In another retrospective cohort of 1443 women with atypical hyperplasia who did not undergo hysterectomy (followed from 1985 to 2005), the incidence of invasive adenocarcinoma over 21 years was 2.9% for hyperplasia without atypia and 14.9% for atypical hyperplasia, with lower progression rates among those treated with progestins. When excluding women diagnosed with carcinoma within one year of hyperplasia diagnosis—likely representing synchronous disease—the mean time to progression was estimated at 5.1 years for non-atypical hyperplasia and 2.5 years for atypical hyperplasia [50]. A recent meta-analysis of 10 studies confirmed that approximately 32% of women with atypical endometrial hyperplasia received a concurrent diagnosis of endometrial cancer. The overall future risk of progression to endometrial cancer for patients with atypical hyperplasia was confirmed to be 8.2% per year [51,52]. Recent studies suggest that progression from atypical hyperplasia/endometrial intraepithelial neoplasia (EIN) to endometrial carcinoma is driven by a complex sequence of molecular alterations, including abnormalities in PTEN, KRAS, PIK3CA, and mismatch repair pathways [53]. Consistent with the growing emphasis on molecular characterization in endometrial neoplasia, several biomarkers are being investigated to improve risk stratification among patients with AH/EIN. Such approaches may enhance the prediction of progression risk and ultimately support more personalized treatment and follow-up strategies [54].

Consensus among major guidelines highlights that total hysterectomy is the preferred treatment for atypical endometrial hyperplasia. The chosen surgical approach should enable adequate evaluation of the adnexa and other pelvic structures to assess for possible invasive disease when indicated. There is currently no evidence guiding the optimal surgical route for hysterectomy in women with endometrial hyperplasia without atypia. The RCOG recommends minimally invasive approaches, as they allow appropriate staging and are associated with shorter hospital stays, reduced postoperative pain, and faster recovery. Similarly, the SOGC and Chinese Guidelines consider all surgical routes acceptable but also favor vaginal or laparoscopic hysterectomy. Guidelines differ in surgical details for atypical endometrial hyperplasia but concur on avoiding certain techniques. In terms of surgical techniques, both RCOG and SOGC, as well as the Chinese Guidelines, discourage uterine morcellation, whereas ACOG allows morcellation only within a contained system to prevent tissue spillage. Moreover, all guidelines agree that subtotal (supracervical) hysterectomy should be avoided, as it precludes assessment of potential cervical stromal involvement. When considering intraoperative assessment, the RCOG, SOGC, and Chinese Guidelines do not recommend frozen-section analysis of the endometrium, citing its limited reliability and inconsistent evidence from small observational studies [55,56,57,58]. In contrast, ACOG supports considering frozen section evaluation during surgery, given the high rate of concurrent endometrial carcinoma, as it may guide the need for additional surgical management.

#### 3.4.1. Bilateral Salpingo-Oophorectomy

All major guidelines concur that peri- and postmenopausal women with atypical endometrial hyperplasia should undergo bilateral salpingo-oophorectomy (BSO) at the time of total hysterectomy. While this recommendation is consistent, the SOGC appears more inclined than others to recommend BSO even in premenopausal women. In contrast, most guidelines emphasize that, in premenopausal women, the decision should involve shared decision-making between the clinician and the patient, given the long-term consequences of surgical menopause. Additionally, the RCOG advises that bilateral salpingectomy may be considered in premenopausal women as a preventive measure to reduce future ovarian cancer risk. Furthermore, for premenopausal women undergoing hysterectomy with BSO for endometrial hyperplasia, RCOG recommends estrogen replacement therapy, in the absence of contraindications, until the expected age of natural menopause to mitigate the adverse effects of estrogen deprivation. Ultimately, all guidelines underscore the importance of individualized management and comprehensive counseling to support informed patient choice.

#### 3.4.2. Sentinel Lymph Node Biopsy and/or Lymphadenectomy

Sentinel lymph node biopsy (SNB) has now largely replaced systematic lymph node dissection and currently represents a valuable diagnostic tool in the surgical staging of endometrial carcinoma; however, its role in endometrial hyperplasia remains controversial. Although concern exists regarding occult malignancy in cases of atypical endometrial hyperplasia, the associated carcinomas are typically early-stage and carry a low risk of lymph vascular involvement [59]. The main challenge is identifying which patients with atypical hyperplasia may harbor or subsequently develop carcinoma and might benefit from SNB in terms of accurate staging and management of adjuvant therapy.

Recently published studies have highlighted the feasibility and safety of the procedure, reporting complication rates comparable to those of hysterectomy alone. Moreover, they have demonstrated a remarkably high prevalence of concurrent endometrial carcinoma in this population, with sentinel lymph node biopsy providing prognostic and therapeutic information, allowing an accurate risk stratification. These findings further support the potential role of incorporating sentinel lymph node mapping into hysterectomy for patients with atypical hyperplasia [60,61]. However, the role of sentinel lymph node mapping in these patients remains uncertain, as current evidence is insufficient to support its routine use outside selected clinical settings or research protocols. Accurate pathological and molecular assessment of endometrial biopsies remains crucial to detect concurrent malignancy [62]. Both SCOG and RCOG, as well as the Chinese Guidelines, agree that lymphadenectomy should not be routinely performed in atypical hyperplasia because this would result in unnecessary surgical risk for most women. ACOG does not mention this topic.

#### 3.4.3. Medical Treatment

Medical therapy is not usually first-line for atypical endometrial hyperplasia (AEH). Still, it may be reasonable for women seeking fertility, those who decline surgery, or who are high-risk for surgery. This is accepted by all major guidelines when comprehensive counseling is provided. Patients must be told about the chance of concurrent carcinoma and the risk of progression to endometrioid adenocarcinoma. Adherence to medical treatment and strict, close follow-up are essential. Goals differ by patient: for those wanting fertility, aim for full disease regression, normal endometrial function, and prevention of invasive disease; for those unfit for surgery, focus on disease control and cancer prevention. Both RCOG and SCOG consider several hormonal therapies, including oral progestogens, the LNG-IUS, aromatase inhibitors, and gonadotropin-releasing hormone agonists. However, the cornerstone of fertility-sparing therapy in AEH remains continuous progestin-based treatment, either orally or via the levonorgestrel-releasing intrauterine system (LNG-IUS). Although no randomized controlled trials have directly compared medical regimens, observational studies have reported favorable outcomes in terms of histological regression and reproductive success. A meta-analysis of observational data found overall rates of disease regression at 85.6%, recurrence at 26%, and a live birth rate of 26.3%. Given the high relapse rate and limited long-term follow-up in primary studies, the authors recommend that a hysterectomy be performed once fertility is no longer desired [63]. The RCOG clearly recommends the levonorgestrel-releasing intrauterine system (LNG-IUS) as the first-line treatment for atypical endometrial hyperplasia, with oral progestogens as a suitable alternative when intrauterine therapy is not feasible. The SOGC provides similar guidance, endorsing the same agents used for non-atypical hyperplasia but proposing adding metformin to enhance therapeutic efficacy, even in women without metabolic syndrome. The ACOG acknowledges the lack of robust comparative evidence between oral and intrauterine progestin administration but reports that intrauterine delivery appears to achieve higher histological regression rates than oral therapy alone. However, it concludes that no single oral formulation can be specifically recommended due to insufficient data, underscoring the need for further well-designed studies [64]. Conversely, the SIGO takes a stronger position in favor of the LNG-IUS, recommending it as the preferred first-line medical treatment, given its higher disease regression rate, more favorable bleeding profile, and lower incidence of adverse effects compared with oral progestins. The ESGO/ESHRE/ESGE guidelines instead propose a combined therapeutic approach if possible. When endometrial hyperplasia is diagnosed, surgical management should include a superficial hysteroscopic endometrial resection that preserves the basal layer of the endometrium, immediately followed by the insertion of a levonorgestrel-releasing intrauterine device (LNG-IUS). This combined, fertility-sparing strategy has shown promising outcomes in both atypical endometrial hyperplasia and early-stage endometrial carcinoma [65]. Regarding molecules, dosage, and regimen, the guidelines recommend the same agents used for endometrial cancer: orally administered megestrol acetate at a dose of 160–320 mg/day or medroxyprogesterone acetate at a dose of 400–600 mg/day. The levonorgestrel intra-uterine device at a dose of 52 mg, alone or in combination with oral progestins, is a safe and effective approach. Italian guidelines recommend using medroxyprogesterone acetate (MPA), megestrol acetate (MA), or an LNG-IUD for at least 3 months (preferably 6) in a continuous regimen. The optimal progestin dose is undefined. Still, recommended oral regimens are MPA 400–600 mg/day or MA 160–320 mg/day. A mention is made by Chinese guidelines regarding the use of gonadotropin-releasing hormone agonist, either alone or in combination with LNG-IUS or aromatase inhibitors. However, there is currently insufficient high-quality evidence to support its effectiveness.

#### 3.4.4. Follow-up

All guidelines agree that regular histologic surveillance is essential during conservative management of atypical endometrial hyperplasia, but they differ in the recommended duration and intensity of follow-up. The RCOG and Chinese Guidelines recommend a minimum treatment duration of 6 months, with an endometrial biopsy every 3 months until 2 consecutive negative results are obtained, followed by 6–12-monthly surveillance. Follow-up should be individualized based on risk factors, particularly obesity, which increases relapse risk, and should include a clinical review, pelvic examination, and biopsy [66]. Transvaginal ultrasound may rule out adnexal disease but is considered not reliable for endometrial evaluation, especially with an LNG-IUS in place. There is no data to support the routine use of MRI or CT during follow-up. In asymptomatic women with a uterus and evidence of histological disease regression, long-term follow-up with endometrial biopsy every 6–12 months is recommended until a hysterectomy is performed. If there is no histologic regression after twelve months or if the disease progresses, hysterectomy should be considered. The highest risk for relapse is within the first two years after diagnosis. Recurrence warrants hysterectomy, as it often coexists with endometrial carcinoma. If surgery is not possible or declined, another round of progestin therapy may be tried. After two years, an annual endometrial biopsy is suggested for asymptomatic women with regression.

The ESHRE/ESGO/ESGE guidelines recommend a 6–12-month course of therapy, noting that most patients achieve regression within 4–6 months. In the absence of any response at 6 months, multidisciplinary counseling is recommended to adapt management on a case-by-case basis; if a complete response is not confirmed within 15 months, hysterectomy is mandatory. Endometrial assessment is advised every 3–6 months by hysteroscopy, with pelvic examination and ultrasound at each visit; MRI may be used selectively. Two consecutive negative biopsies, with a minimal interval of 3 months, are required to confirm remission before transitioning to maintenance or fertility-oriented management. The complete response is mandatory to consider follow-up with maintenance treatment until pregnancy is planned.

The ACOG recommends histologic reassessment every 3–6 months, with the frequency adjusted by factors such as treatment type, menopausal status, and cancer risk profile. Sampling may be performed with the LNG-IUS in situ or after temporary discontinuation of oral progestins. Continued surveillance for up to two years is considered reasonable, after which biopsies may be discontinued in asymptomatic women with sustained remission, though ongoing vigilance for recurrent bleeding is essential. Long-term maintenance progestin therapy may be appropriate for women with persistent risk factors (late menopause, nulliparity, chronic anovulation, Lynch syndrome, Cowden syndrome, obesity, and type 2 diabetes mellitus), but the optimal duration remains undefined.

The Italian Guidelines recommend 3–6 months of treatment, as 12 months do not provide additional benefit. After starting therapy, follow-up evaluations are done every six months using hysteroscopy and endometrial biopsy. This continues until pregnancy, through ART if needed.

If there is no therapeutic response or persistent pathological disease, hysterectomy with bilateral salpingectomy should be advised. Bilateral oophorectomy may be omitted in younger patients (<45 years) without evidence of extrauterine disease. For patients who show a partial response after 6 months of therapy (e.g., complex atypical hyperplasia), continuation of the selected progestin treatment for an additional 3–6 months may be considered.

The SOGC recommends endometrial biopsies every 3 months until 2 consecutive negative specimens are obtained, then every 6 months for 2 years, and annually thereafter until a total hysterectomy is performed. As with RCOG, hysterectomy is indicated in cases of progression, lack of regression after 12 months, relapse, persistent bleeding, or refusal of follow-up. Non-surgical candidates may receive additional cycles of progestin therapy, which remain effective in up to 85% of cases.

Overall, all guidelines stress close endometrial surveillance and individualized management but differ in key ways. RCOG, SOGC, and Chinese Guidelines provide the most structured, long-term follow-up protocols with detailed intervals and indications for hysterectomy. ESHRE/ESGO/ESGE focus on a time-limited treatment approach with strict criteria for complete response, after which maintenance or fertility management is considered. In contrast, ACOG emphasizes ongoing, risk-adapted monitoring and flexible follow-up, with particular attention to individual risk factors and reproductive goals.

#### 3.4.5. Future Fertility

All major international guidelines acknowledge the growing role of fertility-sparing management in selected women with atypical endometrial hyperplasia (AEH) or early-grade endometrioid carcinoma. They consistently emphasize that this approach must be carried out within a controlled, multidisciplinary framework. Thorough counseling on its non-standard nature and potential oncologic risks is necessary. The RCOG highlights the need for careful pre-treatment evaluation to exclude invasive or co-existing ovarian malignancy. It recommends investigations such as CA-125 testing, transvaginal ultrasound, and/or MRI. All results, including histology and imaging, should be discussed in a multidisciplinary team (MDT) before proceeding. Disease regression, confirmed on at least one endometrial sample, is required before attempting conception. Women may try natural conception after remission, but assisted reproductive technology (ART) is encouraged. ART leads to higher live birth rates and lower relapse risk than spontaneous conception. Immediate ART also avoids a long period without progestogen treatment, reducing relapse risk. The RCOG further stresses that hysterectomy should be advised once fertility is no longer desired due to the high recurrence rate of AEH. Similarly, the SOGC recommends early referral to a reproductive endocrinologist, recognizing the risks of subfertility and recurrence in this population. Fertility preservation options include oocyte or embryo cryopreservation, medical therapy followed by ART, or hysterectomy with ovarian preservation for future use of a surrogate. Evidence suggests that infertility treatments do not increase relapse risk, but hysterectomy with bilateral salpingo-oophorectomy (BSO) remains indicated once childbearing is complete [67]. The Italian Guidelines take a structured, highly specialized approach, recommending referral to expert centers for a preliminary fertility assessment and, in selected cases, genetic testing. Diagnosis must be confirmed by two experienced gynaecologic pathologists, ideally supported by immunohistochemical or molecular markers. Patients must consent to intensive follow-up, with hysterectomy recommended in the event of treatment failure, early recurrence, or after a successful pregnancy.

Further, according to the ESGO/ESHRE/ESGE guidelines, women should be encouraged to conceive as soon as a complete histologic response is confirmed, preferably through ART, which improves conception rates and shortens the time to pregnancy without increasing recurrence risk. Natural conception can be considered in selected women with good reproductive potential, but within a definite time (6–9 months). Ongoing multidisciplinary monitoring is essential, and maintenance with an LNG-IUS is advised for women delaying subsequent pregnancies after childbirth or declining surgery.

The ACOG aligns with these views, noting that ART yields better obstetric outcomes than spontaneous conception and that many patients present with comorbidities affecting fertility—such as PCOS, obesity, or diabetes—warranting referral to fertility specialists for proactive management.

In summary, while all guidelines support fertility-sparing treatment in selected cases, RCOG, ESGO/ESHRE/ESGE, and ACOG particularly emphasize ART as the preferred conception strategy, while SOGC and Italian guidelines stress specialist multidisciplinary care and genetic evaluation.

### 3.5. Endometrial Ablation

All major guidelines discourage the use of endometrial ablation in the management of endometrial hyperplasia. The RCOG advises against it because complete and lasting endometrial destruction cannot be guaranteed, and tissue regeneration may occur. In addition, ablation compromises future endometrial surveillance and cannot be considered a fertility-sparing technique [68]. Similarly, the SOGC notes that evidence is insufficient to support ablation as a first-line therapy for hyperplasia without atypia, recommending its use only when major surgery is contraindicated and in consultation with gynaecologic oncology. For atypical hyperplasia, ablation is contraindicated due to its premalignant potential and the difficulty of follow-up in an obliterated endometrial cavity, a particular cause for concern in patients with ongoing risk factors for endometrial carcinoma. The ACOG likewise states that gynecologists should not perform endometrial ablation because of the high persistence and recurrence rates and the potential challenge of assessing subsequent bleeding episodes.

### 3.6. Hormone Replacement Therapy (HRT)

The ACOG, SOGC, as well as Chinese and Italian guidelines, do not specifically address the issue of hormone replacement therapy (HRT) in women with a history of endometrial hyperplasia. Likewise, the RCOG states that continuous combined HRT can be considered safe in women with a prior history of endometrial hyperplasia. Evidence from large observational studies shows no cases of recurrence in women receiving continuous combined HRT and even histologic regression in those who switched from sequential to continuous combined regimens [69,70,71]. Conversely, estrogen-only or sequential HRT preparations should not be used. All women on HRT should be advised to report any unscheduled vaginal bleeding promptly for appropriate investigation. Further studies are required to clarify the impact of modifying or supplementing systemic HRT with locally delivered progestogens via the LNG-IUS and to determine whether combined HRT can be safely resumed after histologic regression of hyperplasia. In contrast, the ESGO/ESHRE/ESGE guidelines comment only on estrogen replacement therapy in women who have undergone premenopausal bilateral salpingo-oophorectomy.

### 3.7. Tamoxifen

The ACOG, SOGC, ESGO/ESHRE/ESGE, Chinese, and Italian guidelines do not specifically recommend how to manage tamoxifen-associated endometrial pathology. In contrast, the RCOG offers detailed guidance distinguishing the risk profiles of tamoxifen and aromatase inhibitors. It emphasizes that women receiving tamoxifen should be informed of the increased risk of endometrial hyperplasia and carcinoma, in addition to being advised to promptly report any abnormal vaginal bleeding or discharge. Meanwhile, women treated with aromatase inhibitors (such as anastrozole, exemestane, or letrozole) should be reassured that these agents are not associated with increased endometrial risk. The likelihood of endometrial pathology with tamoxifen is both dose- and duration-dependent and differs by menopausal status. Evidence from the National Surgical Adjuvant Breast and Bowel Project (P-1) trial demonstrated that the risk of endometrial cancer was not significant in women aged ≤49 years (RR 1.42, 95% CI 0.55–3.81), but significantly higher in women aged ≥50 years (RR 5.33, 95% CI 2.47–13.17). With RCOG guidance established, the recommendations regarding prophylaxis can be considered. Routine progestogen prophylaxis for women on tamoxifen is not recommended. Studies suggest that the levonorgestrel-releasing intrauterine system (LNG-IUS) can reduce the incidence of endometrial polyps and hyperplasia in this population. However, its effect on breast cancer recurrence is uncertain, so its routine use cannot be advised. A 2009 Cochrane review reported that the LNG-IUS significantly reduced the formation of new endometrial polyps. It found no conclusive evidence for preventing hyperplasia or carcinoma. More recent analyses, however, indicate that the LNG-IUS may also reduce hyperplastic changes [72,73].

A randomized trial assessing prophylactic LNG-IUS insertion before tamoxifen therapy confirmed its benefit in preventing polyp formation over five years, but its impact on preventing hyperplasia was unclear, as no cases were observed in either group. Importantly, there was no statistically significant increase in breast cancer recurrence (17.2% vs. 10.0%) or cancer-related mortality (10.3% vs. 8.3%), although the study lacked sufficient power [74].

If endometrial hyperplasia develops during tamoxifen therapy, clinicians should re-evaluate the continued need for tamoxifen in consultation with the patient’s oncologist and manage the condition according to histologic subtype, following the same principles applied to women with hyperplasia unrelated to tamoxifen exposure.

### 3.8. Hyperplasia on Endometrial Polyp

The RCOG and SOGC guidelines are the only ones that specifically address this topic. Both agree that when endometrial hyperplasia is identified within a polyp, the lesion should be completely excised, and subsequent management should be guided by the histologic classification of the hyperplasia. They also emphasize the importance of sampling the background endometrium, even when it appears normal on hysteroscopy, as in the absence of background hyperplasia, polypectomy alone may be curative. Both guidelines cite a small case series comparing LNG-IUS versus no adjuvant treatment after polyp removal with focal atypical hyperplasia, which reported no recurrence of atypia after 5 years in either group [75]. The SOGC additionally cites a systematic review of ten predominantly retrospective studies showing that concurrent endometrial carcinoma occurs in approximately 5.6% of patients with an atypical endometrial polyp [76]. Nevertheless, both acknowledge that evidence remains limited to guide optimal management in this setting.

### 3.9. Evidence Gaps

Despite substantial agreement among contemporary guidelines, several areas of uncertainty remain in the management of endometrial hyperplasia. Significant heterogeneity persists regarding surveillance intervals, treatment duration, criteria for treatment escalation, and the optimal follow-up strategy after histological regression. The management of asymptomatic endometrial thickening, particularly in postmenopausal women and tamoxifen users, also remains supported by limited evidence. Furthermore, although fertility-sparing approaches have shown promising outcomes, high-quality comparative studies evaluating the optimal therapeutic regimen, duration of treatment, reproductive outcomes, and long-term oncologic safety remain lacking. Emerging molecular biomarkers and digital pathology tools may improve risk stratification and diagnostic reproducibility in the future, but their integration into routine clinical practice has not yet been established. Finally, the role of sentinel lymph node mapping in patients with atypical hyperplasia/endometrial intraepithelial neoplasia (EIN) remains investigational, and further prospective studies are required before its routine implementation can be recommended. These evidence gaps likely contribute to the differences observed among current international guidelines and represent important priorities for future research and guideline harmonization.

## 4. Conclusions

**Figure 1:**
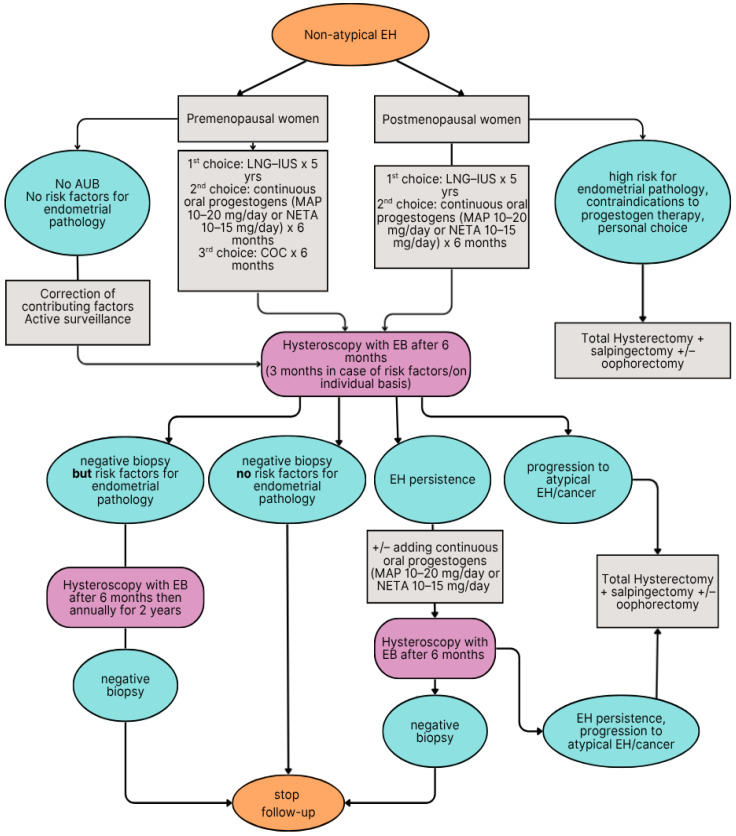
Flow chart 1: evidence-based management approach to non-atypical endometrial hyperplasia. AUB: abnormal uterine bleeding; EH: endometrial hyperplasia; LNG-IUS: levonorgestrel intra-uterine system; MAP: medroxyprogesterone acetate; NETA: norethisterone acetate; COC: continuous oral contraception; EB: endometrial biopsy.

**Figure 2:**
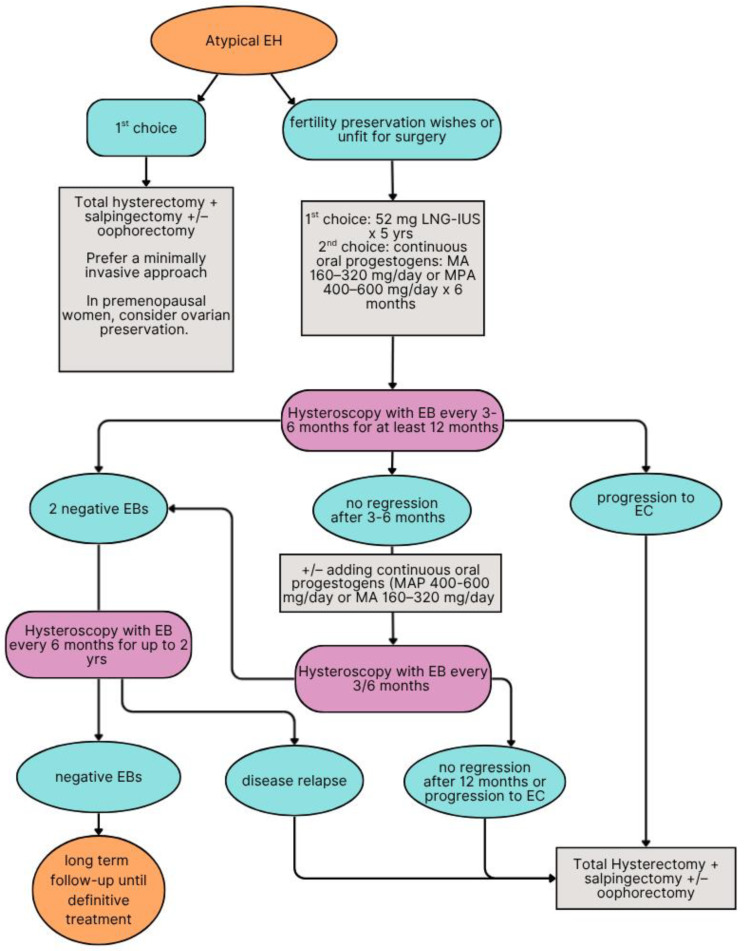
Flow chart 2: evidence-based management approach to atypical endometrial hyperplasia. EH: endometrial hyperplasia; LNG-IUS: levonorgestrel intra-uterine system; MAP: medroxyprogesterone acetate; MA: megestrol acetate; EB: endometrial biopsy; EC: endometrial cancer.

This review highlights substantial concordance among international guidelines on the principles guiding the management of endometrial hyperplasia, including the central role of histologic diagnosis, individualized risk assessment, and alignment of treatment with reproductive goals. Nonetheless, important differences remain in recommended diagnostic techniques, therapeutic strategies, and follow-up protocols, particularly for atypical disease and fertility-sparing approaches. These discrepancies largely reflect heterogeneity in available evidence and differences across health care systems, which can hinder interprofessional comparability and standardization. Considering all this, this study also provides a concise operational proposal presented as practical flow charts, with the aim of translating guideline recommendations into an immediately applicable clinical decision pathway. The proposed flowcharts (section: figures; flow Figure 1 and Figure 2) represent a synthesis of the most consistent recommendations identified across the reviewed guidelines and may support clinical decision-making, while acknowledging that local resources, healthcare infrastructure, and regional practice patterns may influence their implementation. An emerging area of interest is the role of molecular and biomarker-based risk stratification in patients with atypical hyperplasia/endometrial intraepithelial neoplasia (EIN). Alterations involving PTEN, KRAS, PIK3CA, CTNNB1, and mismatch repair pathways have been implicated in the development and progression of endometrial neoplasia. However, despite the growing body of evidence supporting the biological and prognostic significance of these molecular alterations, their integration into routine clinical management has not yet been incorporated into most international guidelines for endometrial hyperplasia. Current recommendations continue to rely primarily on histopathological classification and clinical factors. Future studies may clarify the role of molecular biomarkers in identifying patients at increased risk of progression and in supporting more individualized management strategies. Research should also further explore the role of digital pathology and artificial intelligence-based tools, which have the potential to improve diagnostic reproducibility, reduce interobserver variability, and integrate histologic, immunohistochemical, and molecular features into a more standardized model of care [77,78].

## Figures and Tables

**Table 1:** Diagnostic assessment.

Topic	ACOG [14]	RCOG [15]	SOGC [16]	Chinese [17]	GISS-SIGO [18]	ESGO-ESHRE [19]
**Terminology**	WHO 2020 [11]	WHO 2020 [11]	WHO 2020 [11]	WHO 2020 [11]	WHO 2020 [11]	WHO 2020 [11]
**Endometrial** **sampling**	Hysteroscopic-guided sampling.	Hysteroscopic-guided sampling, especially if outpatient failed/or nondiagnostic. (Level 2+)	1st option: blind approaches (e.g., Pipelle), hysteroscopic guided sampling and curettage if non-diagnostic/highly suspicious, persistent bleeding, cervical stenosis, failed endometrial biopsy, or excessive pain/anxiety	D&C. (Level 2++) or Hysteroscopic endometrial biopsy (level 2+).	N.M.	Hysteroscopically guided endometrial biopsy is preferred over a blind biopsy.
**Imaging** **techniques**	N.M.	There is insufficient evidence to routinely recommend CT or MRI.	N.M.	MRI can aid in the identification of invasive carcinomas.	MRI may be requested to assess potential local disease extension.	N.M.

*Note*: WHO: World Health Organization; D&C: dilation and curettage; CT: computed tomography; MRI: magnetic resonance imaging; N.M: not mentioned.

**Table 2:** Management of non-atypical endometrial hyperplasia.

Topic	ACOG [14]	RCOG [15]	SOGC [16]	Chinese [17]	**GISS-SIGO** [18]	**ESGO-ESHRE** [19]
**Addressing risk factors**	N.M.	Reversible risk factors such as obesity and HRT should be identified and addressed.	Patients should be assessed for reversible risk factors and receive support to treat those conditions.	Patients should be advised to address modifiable risk factors.	N.M.	N.M.
**Medical treatment**	N.M.	1st choice: LNG-IUS (Level 2+) 2nd choice: continuous oral progestogens (MAP 10–20 mg/day or NETA 10–15 mg/day) (Level 1+) Cyclical progestogens should not be used. (Level 1+)	1st choice: LNG-IUS (possibly kept in place for 5 years in patients showing treatment response). 2nd choice oral or injectable progestogens: MPA 100–200 mg/day (high dose) or 2.5–20 mg/day (low dose) MPA 10–20 mg/day per 10–12 days per cycle MPA injectable 150 mg every 90 days MA 80–320 mg/day (high dose) or 40 mg/day (low dose) NETA 5–15 mg/day (continuous) or 15 mg/day per 10–12 days per cycle Progesterone 100–300 mg/day In both pre- and post-menopausal patients	1st choice: LNG-IUS (meta evidence level 1−, RCT evidence level 2+) 2nd choice: Oral progestogen. MPA 10–20 mg/day MA 40 mg/day dydrogesterone 20 mg/day NETA 15 mg/day Other drugs as off-label options: COC, aromatase inhibitors, GnRH agonist.	N.M.	N.M.
**Duration**	N.M.	Treatment duration (with either oral progestogens or the LNG-IUS) should last a minimum of 6 months. If adverse effects are tolerable and fertility is not desired, women should be encouraged to retain the LNG-IUS for up to 5 years, as this reduces the risk of relapse.	Treatment duration (with either oral progestogens or the LNG-IUS) should last a minimum of 6 months The LNG-IUS should be kept in place for 5 years in patients showing a treatment response.	Treatment duration should last a minimum of 6 months. If adverse effects are tolerable and fertility is not desired, women should be encouraged to retain the LNG-IUS for up to 5 years, as this reduces the risk of relapse.	N.M.	N.M.
**Follow-up**	N.M.	Endometrial sampling should be arranged at least every 6 months, but timing should be individualized. At least two consecutive 6-monthly negative biopsies should be obtained prior to discharge. In women at higher risk of relapse (like a BMI of 35 kg/m^2^ or greater), 6-monthly endometrial biopsies are recommended. Once two consecutive negative biopsies are obtained, long-term follow-up should be considered with annual endometrial biopsies. (Level 2+)	Endometrial sampling (performed with the IUD in place), every 3 to 6 months. The decision to continue therapy beyond 6 months for patients who do not respond should be made on an individual basis. A response after 12 months of treatment will rarely occur, and a change in treatment modality should be considered. The follow-up duration for patients with risk factors (like a BMI of 35 kg/m^2^ or greater) should be extended, as the relapse rate has proven to be higher.	Endometrial sampling every 6 months. At least two consecutive 6-monthly negative biopsies should be obtained prior to discharge. Then, clinical and TV-US follow-up once a year.	N.M.	N.M.
**Surgery indications**	N.M.	Hysterectomy should be considered if patient does not want fertility preservation there is progression to AH or carcinoma during follow-up hyperplasia fails to regress after 12 months of medical treatment hyperplasia relapses after completing treatment abnormal uterine bleeding continues despite treatment patient declines surveillance medical treatment is contraindicated	Hysterectomy should be considered if: patient does not want fertility preservation there is progression to AH or carcinoma during follow-up hyperplasia fails to regress after 12 months of medical treatment hyperplasia relapses after completing treatment abnormal uterine bleeding continues despite treatment patient declines surveillance medical treatment is contraindicated	Hysterectomy should be considered if there is progression to AH or carcinoma during follow-up hyperplasia fails to regress after 12 months of medical treatment hyperplasia relapses after completing treatment abnormal uterine bleeding continues despite treatment patient declines surveillance medical treatment is contraindicated	N.M.	N.M.
**Type of surgery**	N.M.	Postmenopausal women should be offered a BSO together with a total hysterectomy. For premenopausal women, the decision to remove the ovaries should be individualized; bilateral salpingectomy should be considered, as this may reduce the risk of a future ovarian malignancy. A laparoscopic approach is preferable. (Level 1+) Endometrial ablation is not recommended. (Level 1−)	Surgical treatment should consist of total hysterectomy with salpingectomy, with or without bilateral oophorectomy. The ovaries are typically removed in postmenopausal women. Prophylactic salpingectomy alone at the time of hysterectomy can be offered for risk reduction in ovarian cancer when the ovaries are left in situ. A minimally invasive approach is preferred.	TH. Subtotal hysterectomy is not appropriate. There is no indication of sentinel lymph node biopsy or lymphadenectomy. Endometrial ablation is not recommended.	N.M.	N.M.

*Note:* HRT: hormone replacement therapy; LNG-IUS: levonorgestrel intrauterine system; MA: megestrol acetate; MPA: medroxyprogesterone acetate; NETA: norethindrone acetate; N.M: not mentioned; TH: total hysterectomy; BSO: bilateral salpingo-oophorectomy, TV-US: transvaginal ultrasound, COC: combined oral contraceptive.

**Table 3:** Management of atypical endometrial hyperplasia.

Topic	ACOG [14]	RCOG [15]	SOGC [16]	Chinese [17]	**GISS-SIGO** [18]	**ESGO-ESHRE** [19]
**Surgery**	TH is the definitive treatment. Avoid subtotal hysterectomy. Endometrial ablation is not recommended	TH is the standard treatment. (Level 1+) A minimally invasive approach is preferable. Postmenopausal women with atypical hyperplasia should be offered BSO together with the TH. (Good practice point) In premenopausal women, bilateral salpingectomy must be considered. Bilateral oophorectomy should be individualized. (Level 2++) Endometrial ablation is not recommended (Level 2+) No benefit from routine intraoperative frozen section analysis or routine lymphadenectomy. (Level 2+)	TH with BSO is the standard treatment. In premenopausal women, ovarian preservation should be discussed. Avoid subtotal hysterectomy and uterine morcellation. Endometrial ablation is not recommended No benefit from routine intraoperative frozen section analysis or routine lymphadenectomy.	Postmenopausal women should be offered BSO together with TH. For premenopausal women, salpingectomy should be considered as this may reduce the risk of a future ovarian malignancy. No benefit from routine intraoperative frozen section analysis or routine lymphadenectomy	N.M.	N.M.
**Medical treatment**	For patients in whom TH is not an option, progestins are recommended. LNG-IUD seems to be associated with higher rates of regression compared to oral formulations. There is insufficient evidence to recommend any oral formulation over another.	If a patient wishes to preserve their fertility or is unfit for surgery. First-line treatment with the LNG-IUS, with oral progestogens as a second-best alternative. (Level 2++).	If a patient wishes to preserve their fertility or is unfit for surgery. Conservative options include: 52 mg LNG-IUD MPA 100–200 mg/day (high dose) or 2.5–20 mg/day (low dose) MPA 10–20 mg/day per 10–12 days per cycle MPA injectable 150 mg every 90 days MA 80–320 mg/day (high dose) or 40 mg/day (low dose) NETA 5–15 mg/day (continuous) or 15 mg/day per 10–12 days per cycle Progesterone 100–300 mg/da aromatase inhibitors GnRh agonists Metformin may be added to increase treatment effect, even in the absence of metabolic syndrome. Weight loss should be encouraged.	If a patient wishes to preserve their fertility or is unfit for surgery, after counseling. Drug regimens: 52 mg LNG-IUS (evidence level 2+) MA 160 mg once/twice per day MPA 500 mg/day For patients without short-term fertility requirements, placement of an LNG-IUS, the use of oral progestins, or short-acting contraceptives is recommended to protect the endometrium and prevent recurrence (evidence level 2+).	Continuous oral MA 160–320 mg/day or MPA 400–600 mg/day and/or 52 mg LNG-IUD for at least 3–6 months (IUD preferred). No clear advantages in extending treatment to 12 months.	Combined hysteroscopic resection with progestin therapy is the most effective fertility-sparing treatment. Oral MA 160–320 mg/day or MPA 400–600 mg/day in combination with a 52 mg LNG-IUD is the best option. The recommended duration of medical therapy is 6–12 months. The maximum time to achieve a complete response should not exceed 15 months. In the absence of any kind of response at 6 months, multidisciplinary counseling is recommended for adapting the management on a case-by-case basis. Weight control is highly recommended to increase the chance of response.
**Follow-up**	Endometrial surveillance every 3–6 months. If there is no regression after 3–6 months, an additional 3–6 months of therapy may be considered. If there is no regression in 9–12 months, definitive surgery must be discussed. After a complete response, it is reasonable to continue endometrial sampling every 3–6 months up to 2 years.	Endometrial surveillance every 3 months until at least 2 negative specimens are obtained. (Level 4) After disease regression, long-term follow-up with an endometrial biopsy every 6–12 months is recommended until TH is performed. (Level 2++)	Endometrial surveillance every 3 months until at least 2 negative specimens are obtained. After treatment cessation, an endometrial biopsy should be performed every 6 months for 2 years and then every year until risk factors are corrected or TH with BSO is performed. Definitive surgery if: progression to carcinoma disease persistence after 12 months of medical treatment disease relapse after completing treatment persistent AUB scarce compliance with surveillance or medical treatment If unfit for surgery, a 2nd and 3rd round of progestins are an option.	Endometrial surveillance every 3 months during treatment until at least 2 negative specimens are obtained. Treatment dose or regimen should be adjusted according to the level of drug response (evidence level 2++) After disease regression, endometrial biopsy every 6–12 months until risk factors are removed, or until a total hysterectomy is performed. Definitive surgery if persistence or progression after 12 months of standard treatment, recurrence after completing treatment, and no desire for fertility, persistent abnormal uterine bleeding, or an inability to follow up or adhere to medication.	Hysteroscopic endometrial biopsy should be performed at least every 6 months until pregnancy is achieved (also through ART). In the absence of a pathological response, hysterectomy with bilateral salpingectomy is indicated. Oophorectomy may be omitted. Once pregnancy is achieved, TH with BS is recommended. Oophorectomy may be omitted.	Clinical pelvic examination and US are recommended at every 3-month follow-up visit. Endometrial sampling should be performed every 3–6 months by hysteroscopy. MRI could be considered on a case-by-case basis. Two consecutive endometrial biopsies showing complete response with a minimal interval of 3 months are necessary. The complete response is mandatory to consider follow-up with maintenance treatment until pregnancy is planned.
**Fertility issues**	Active management to achieve pregnancy should be discussed, and referral to an infertility specialist should be considered	Women wishing to retain their fertility should receive adequate counseling. Disease regression should be achieved on at least one endometrial sample before women attempt to conceive. (Level 4) Women should be referred to a fertility specialist. ART should be considered to improve the success rate and reduce the interval to conception. (Level 2++) Once fertility is no longer required, TH should be offered (Level 2++)	Patients should be referred to a reproductive center for counseling/ART. TH with BSO is recommended when child- bearing is no longer desired	Patients with fertility requirements should be advised to try to get pregnant after complete remission of the disease, preferably with assisted reproductive technology (evidence level 3).	N.M.	Women should be encouraged to actively aim to conceive as soon as the complete response is achieved. ART should be considered to improve the success rate and reduce the interval to conception. Natural conception may be considered in women with good reproductive potential within 6–9 months. Close surveillance by a multidisciplinary team and maintenance therapy with LNG-IUD is recommended to women who decline surgery after delivery or do not plan a 2nd pregnancy immediately.

*Note:* LNG-IUS: levonorgestrel intrauterine system; MA: megestrol acetate; MPA: medroxyprogesterone acetate; NETA: norethindrone acetate; N.M: not mentioned. TH: total hysterectomy; BSO: bilateral salpingo-oophorectomy.

## Data Availability

Not applicable.

## References

[1] Singh G., Cue L., Puckett Y. (2025). Endometrial Hyperplasia. StatPearls.

[2] Emons G., Beckmann M.W., Schmidt D., Mallmann P. (2015). Uterus commission of the Gynecological Oncology Working Group. New WHO classification of endometrial hyperplasias. Geburtsh. Frauenheilk..

[3] Moshayedi N., Clair K., Francoeur A.A. (2025). Obesity management in the setting of endometrial cancer and hyperplasia: A narrative review. Gynecol. Oncol. Rep..

[4] Agarwal R., Stanczyk F.Z., Mandelbaum R., Paulson R.J., McGinnis L.K., Winer S.A. (2025). Prevalence of endometrial hyperplasia and carcinoma in women with polycystic ovarian syndrome. F&S Rep..

[5] Ryu K.J., Kim M.S., Lee J.Y., Nam S., Jeong H.G., Kim T., Park H. (2022). Risk of Endometrial Polyps, Hyperplasia, Carcinoma, and Uterine Cancer After Tamoxifen Treatment in Premenopausal Women With Breast Cancer. JAMA Netw. Open.

[6] Zhou X., Xu Y., Yin D., Zhao F., Hao Z., Zhong Y., Zhang J., Zhang B., Yin X. (2020). Type 2 diabetes mellitus facilitates endometrial hyperplasia progression by activating the proliferative function of mucin O-glycosylating enzyme GALNT2. BioMed. Pharmacother..

[7] Karstensen S., Jochumsen K., Hogdall C., Hogdall E., Marcussen N., Lauszus F.F. (2025). Ovarian sex cord-stromal cell tumors and the risk of sex hormone-sensitive cancers. Am. J. Obstet. Gynecol..

[8] Bowen M.B., Melendez B., Zhang Q., Moreno D., Peralta L., Chan W.K., Jeter C., Tan L., Zal M.A., Lorenzi P.L. (2025). Mitochondrial defects and metabolic vulnerabilities in Lynch syndrome-associated MSH2-deficient endometrial cancer. JCI Insight.

[9] Zhao S., Chen L., Zang Y., Liu W., Liu S., Teng F., Xue F., Wang Y. (2022). Endometrial cancer in Lynch syndrome. Int. J. Cancer.

[10] Niskakoski A., Pasanen A., Porkka N., Eldfors S., Lassus H., Renkonen-Sinisalo L., Kaur S., Mecklin J.P., Bützow R., Peltomäki P. (2018). Converging endometrial and ovarian tumorigenesis in Lynch syndrome: Shared origin of synchronous carcinomas. Gynecol. Oncol..

[11] Kurman R.J., Carcangiu M.L., Herrington C.S., Young R.H. (2014). WHO Classification of Tumours of Female Reproductive Organs.

[12] Herrington C.S. (2020). WHO Classification of Tumours Female Genital Tumours.

[13] Mutter G.L., Baak J.P., Crum C.P., Richart R.M., Ferenczy A., Faquin W.C. (2000). Endometrial precancer diagnosis by histopathology, clonal analysis, and computerized morphometry. J. Pathol..

[14] American College of Obstetricians and Gynecologists (2023). Management of Endometrial Intraepithelial Neoplasia or Atypical Endometrial Hyperplasia: ACOG Clinical Consensus No. 5. Obstet. Gynecol..

[15] Royal College of Obstetricians and Gynaecologists, British Society for Gynaecological Endoscopy (2016). Management of endometrial hyperplasia. RCOG BSGE Jt. Guidel..

[16] Auclair M.H., Yong P.J., Salvador S., Thurston J., Colgan T.T.J., Sebastianelli A. (2019). Guideline No. 390-Classification and Management of Endometrial Hyperplasia. J. Obstet. Gynaecol. Can..

[17] Li L., Zhu L. (2024). Group for Chinese Guidelines on the Management of Endometrial Hyperplasia. Chinese guidelines on the management of endometrial hyperplasia. Eur. J. Surg. Oncol..

[18] SIGO, AGUI, AOGOI, AGITE in Collaborazione con SIOG (2023). Terapia Conservativa in Caso di Tumore Endometrio Stadio IA di Tipo Endometrioide o Iperplasia Endometriale. https://www.aogoi.it/linee-guida/altre-raccomandazioni/terapia-conservativa-tumore-endometrio/.

[19] Rodolakis A., Scambia G., Planchamp F., Acien M., Di Spiezio Sardo A., Farrugia M., Grynberg M., Pakiz M., Pavlakis K., Vermeulen N. (2023). ESGO/ESHRE/ESGE Guidelines for the fertility-sparing treatment of patients with endometrial carcinoma. Hum. Reprod. Open.

[20] (2015). Committee on Gynecologic Practice, Society of Gynecologic Oncology. The American College of Obstetricians and Gynecologists Committee Opinion no. 631. Endometrial intraepithelial neoplasia. Obstet. Gynecol..

[21] Travaglino A., Raffone A., Saccone G., Mollo A., De Placido G., Insabato L., Zullo F. (2019). Endometrial hyperplasia and the risk of coexistent cancer: WHO versus EIN criteria. Histopathology.

[22] Vitale S.G., Buzzaccarini G., Riemma G., Pacheco L.A., Di Spiezio Sardo A., Carugno J., Chiantera V., Török P., Noventa M., Haimovich S. (2023). Endometrial biopsy: Indications, techniques and recommendations. An evidence-based guideline for clinical practice. J. Gynecol. Obstet. Hum. Reprod..

[23] Zhang L., Guo Y., Qian G., Su T., Xu H. (2022). Value of endometrial thickness for the detection of endometrial cancer and atypical hyperplasia in asymptomatic postmenopausal women. BMC Womens Health.

[24] Giri S.K., Nayak B.L., Mohapatra J. (2021). Thickened Endometrium: When to Intervene? A Clinical Conundrum. J. Obstet. Gynaecol. India.

[25] Wang J., Peng X., Xia E. (2024). When is it necessary to perform biopsy in asymptomatic postmenopausal women with incidental finding of thickened endometrium?. Eur. J. Obstet. Gynecol. Reprod. Biol..

[26] Kamińska A.I., Piecak K., Milart P., Czuczwar P., Paszkowski T. (2025). Tamoxifen treatment in breast cancer: Diagnostic methods for endometrial changes. Prz. Menopauzalny.

[27] Clark T.J., Mann C.H., Shah N., Khan K.S., Song F., Gupta J.K. (2002). Accuracy of outpatient endometrial biopsy in the diagnosis of endometrial cancer: A systematic quantitative review. BJOG.

[28] Clark T.J., Mann C.H., Shah N., Khan K.S., Song F., Gupta J.K. (2001). Accuracy of outpatient endometrial biopsy in the diagnosis of endometrial hyperplasia. Acta Obstet. Gynecol. Scand..

[29] Dijkhuizen F.P., Mol B.W., Brolmann H.A., Heintz A.P.M. (2000). The accuracy of endometrial sampling in the diagnosis of patients with endometrial carcinoma and hyperplasia. Cancer.

[30] Kurman R.J., Kaminski P.F., Norris H.J. (1985). The behavior of endometrial hyperplasia. A long-term study of “untreated” hyperplasia in 170 patients. Cancer.

[31] Lacey J.V., Sherman M.E., Rush B.B., Ronnett B.M., Ioffe O.B., Duggan M.A., Glass A.G., Richesson D.A., Chatterjee N., Langholz B. (2010). Absolute risk of endometrial carcinoma during 20-year follow-up among women with endometrial hyperplasia. J. Clin. Oncol..

[32] Nees L.K., Heublein S., Steinmacher S., Juhasz-Böss I., Brucker S., Tempfer C.B., Wallwiener M. (2022). Endometrial hyperplasia as a risk factor of endometrial cancer. Arch. Gynecol. Obstet..

[33] Prip C.M., Stentebjerg M., Bennetsen M.H., Petersen L.K., Bor P. (2022). Risk of atypical hyperplasia and endometrial carcinoma after initial diagnosis of non-atypical endometrial hyperplasia: A long-term follow-up study. PLoS ONE.

[34] Argenta P.A., Kassing M., Truskinovsky A.M., Svendsen C.A. (2013). Bariatric surgery and endometrial pathology in asymptomatic morbidly obese women: A prospective, pilot study. BJOG.

[35] Modesitt S.C., Hallowell P.T., Slack-Davis J.K., Michalek R.D., Atkins K.A., Kelley S.L., Arapovic S., Shupnik M.A., Hoehn K. (2015). Women at extreme risk for obesity-related carcinogenesis: Baseline endometrial pathology and impact of bariatric surgery on weight, metabolic profiles and quality of life. Gynecol. Oncol..

[36] Bandera E.V., Williams M.G., Sima C., Bayuga S., Pulick K., Wilcox H., Soslow R., Zauber A.G., Olson S.H. (2009). Phytoestrogen consumption and endometrial cancer risk: A population-based case–control study in New Jersey. Cancer Causes Control.

[37] Schumer S.T., Cannistra S.A. (2003). Granulosa cell tumor of the ovary. J. Clin. Oncol..

[38] Gallos I.D., Shehmar M., Thangaratinam S., Papapostolou T.K., Coomarasamy A., Gupta J.K. (2010). Oral progestogens vs levonorgestrel-releasing intrauterine system for endometrial hyperplasia: A systematic review and metaanalysis. Am. J. Obstet. Gynecol..

[39] Nilsson C.G., Haukkamaa M., Vierola H., Luukkainen T., Arcangeli P. (1982). Tissue concentrations of levonorgestrel in women using a levonorgestrel-releasing IUD. Clin. Endocrinol..

[40] Abu Hashim H., Ghayaty E., El Rakhawy M. (2015). Levonorgestrel-releasing intrauterine system vs oral progestins for non-atypical endometrial hyperplasia: A systematic review and metaanalysis of randomized trials. Am. J. Obstet. Gynecol..

[41] Ozdegirmenci O., Kayikcioglu F., Bozkurt U., Akqul M.A., Haberal A. (2011). Comparison of the efficacy of three progestins in the treatment of simple endometrial hyperplasia without atypia. Gynecol. Obstet. Investig..

[42] Rattanachaiyanont M., Angsuwathana S., Techatrisak K., Tanmahasamut P., Indhavivadhana S., Leerasiri P. (2005). Clinical and pathological responses of progestin therapy for non-atypical endometrial hyperplasia: A prospective study. J. Obstet. Gynaecol. Res..

[43] Nooh A.M., Abdeldayem H.M., Girbash E.F., Arafa E.M., Atwa K., Abdel-Raouf S.M. (2016). Depo-Provera versus norethisterone acetate in management of endometrial hyperplasia without atypia. Reprod. Sci..

[44] Moradan S., Nikkhah N., Mirmohammadkhanai M. (2017). Comparing the administration of letrozole and megestrol acetate in the treatment of women with simple endometrial hyperplasia without atypia: A randomized clinical trial. Adv. Ther..

[45] Dolapcioglu K., Boz A., Baloglu A. (2013). The efficacy of intrauterine versus oral progestin for the treatment of endometrial hyperplasia. A prospective randomized comparative study. Clin. Exp. Obstet. Gynecol..

[46] Parker W.H., Broder M.S., Liu Z., Shoupe D., Farquhar C., Berek J.S. (2005). Ovarian conservation at the time of hysterectomy for benign disease. Obstet. Gynecol..

[47] Parker W.H. (2010). Bilateral oophorectomy versus ovarian conservation: Effects on long-term women’s health. J. Minim. Invasive Gynecol..

[48] Rakha E., Wong S.C., Soomro I., Chaudry Z., Sharma A., Deen S., Chan S., Abu J., Nunns D., Williamson K. (2012). Clinical outcome of atypical endometrial hyperplasia diagnosed on an endometrial biopsy: Institutional experience and review of literature. Am. J. Surg. Pathol..

[49] Matsuo K., Ramzan A.A., Gualtieri M.R., Mhawech-Fauceglia P., Machida H., Moeini A., Dancz C.E., Ueda Y., Roman L.D. (2015). Prediction of concurrent endometrial carcinoma in women with endometrial hyperplasia. Gynecol. Oncol..

[50] Reed S.D., Newton K.M., Garcia R.L., Allison K.H., Voigt L.F., Jordan C.D., Epplein M., Swisher E., Upson K., Ehrlich K.J. (2010). Complex hyperplasia with and without atypia: Clinical outcomes and implications of progestin therapy. Obstet. Gynecol..

[51] Chou A.J., Bing R.S., Ding D.C. (2024). Endometrial Atypical Hyperplasia and Risk of Endometrial Cancer. Diagnostics.

[52] Doherty M.T., Sanni O.B., Coleman H.G., Cardwell C.R., McCluggage W.G., Quinn D., Wylie J., McMenamin Ú.C. (2020). Concurrent and future risk of endometrial cancer in women with endometrial hyperplasia: A systematic review and meta-analysis. PLoS ONE.

[53] Li L., Yue P., Song Q., Yen T.-T., Asaka S., Wang T.-L., Beavis A.L., Fader A.N., Jiao Y., Yuan G. (2021). Genome-Wide Mutation Analysis in Precancerous Lesions of Endometrial Carcinoma. J. Pathol..

[54] Sanderson P.A., Esnal-Zufiaurre A., Arends M.J., Herrington C.S., Collins F., Williams A.R.W., Saunders P.T.K. (2022). Improving the Diagnosis of Endometrial Hyperplasia Using Computerized Analysis and Immunohistochemical Biomarkers. Front. Reprod. Health.

[55] Boyraz G., Basaran D., Salman M.C., Ozgul N., Yuce K. (2016). Does preoperative diagnosis of endometrial hyperplasia necessitate intraoperative frozen section consultation?. Balk. Med. J..

[56] Stephan J.M., Hansen J., Samuelson M., McDonald M., Chin Y., Bender D., Reyes H.D., Button A., Goodheart M.J. (2014). Intra-operative frozen section results reliably predict final pathology in endometrial cancer. Gynecol. Oncol..

[57] Montalto S.A., Coutts M., Devaja O., Summers J., Jyothirmayi R., Papadopoulos A. (2008). Accuracy of frozen section diagnosis at surgery in pre- malignant and malignant lesions of the endometrium. Eur. J. Gynaecol. Oncol..

[58] Oz M., Ozgu E., Korkmaz E., Bayramoglu H., Erkaya S., Gungor T. (2014). Utility of frozen section pathology with endometrial pre-malignant lesions. Asian Pac. J. Cancer Prev..

[59] Vieira-Serna S., Peralta J., Viveros-Carreño D., Rodriguez J., Feliciano-Alfonso J.E., Pareja R. (2023). Sentinel lymph node assessment in patients with atypical endometrial hyperplasia: A systematic review and meta-analysis. Int. J. Gynecol. Cancer.

[60] Restaino S., Poli A., Arcieri M., Mariuzzi L., Orsaria M., Tulisso A., Paparcura F., Pellecchia G., Petrillo M., Capobianco G. (2025). Is there a role for the sentinel lymph node in endometrial atypical hyperplasia? Insights from an ESGO-accredited Institution. Eur. J. Surg. Oncol..

[61] Rosati A., Vargiu V., Capozzi V.A., Giannarelli D., Palmieri E., Baroni A., Perrone E., Berretta R., Cosentino F., Scambia G. (2024). Concurrent endometrial cancer in atypical endometrial hyperplasia and the role of sentinel lymph nodes: Clinical insights from a multicenter experience. Int. J. Gynecol. Cancer.

[62] Billone V., De Paola L., Conti E., Borsellino L., Kozinszky Z., Giampaolino P., Suranyi A., Della Corte L., Andrisani A., Cucinella G. (2025). Sentinel Lymph Node in Endometrial Hyperplasia: State of the Art and Future Perspectives. Cancers.

[63] Gallos I.D., Yap J., Rajkhowa M., Luesley D.M., Coomarasamy A., Gupta J.K. (2012). Regression, relapse, and live birth rates with fertility-sparing therapy for endometrial cancer and atypical complex endometrial hyperplasia: A systematic review and metaanalysis. Am. J. Obstet. Gynecol..

[64] Fang F., Xu H., Wu L., Hu L., Liu Y., Li Y., Zhang C. (2021). LNG-IUS combined with progesterone ameliorates endometrial thickness and pregnancy outcomes of patients with early-stage endometrial cancer or atypical hyperplasia. Am. J. Transl. Res..

[65] Giampaolino P., Di Spiezio Sardo A., Mollo A., Raffone A., Travaglino A., Boccellino A., Zizolfi B., Insabato L., Zullo F., De Placido G. (2019). Hysteroscopic endometrial focal resection followed by levonorgestrel intrauterine device insertion as a fertility-sparing treatment of atypical endometrial hyperplasia and early endometrial cancer: A retrospective study. J. Minim. Invasive Gynecol..

[66] Penner K.R., Dorigo O., Aoyama C., Ostrzega N., Balzer B.L., Rao J., Walsh C.S., Cass I., Holschneider C.H. (2012). Predictors of resolution of complex atypical hyperplasia or grade 1 endometrial adenocarcinoma in premenopausal women treated with progestin therapy. Gynecol. Oncol..

[67] Ichinose M., Fujimoto A., Osuga Y., Minaguchi T., Kawana K., Yano T., Kozuma S. (2013). The influence of infertility treatment on the prognosis of endometrial cancer and atypical complex endometrial hyperplasia. Int. J. Gynecol. Cancer.

[68] Edris F., Vilos G.A., Al-Mubarak A., Ettler H.C., Hollett-Caines J., Abu-Rafea B. (2007). Resectoscopic surgery may be an alternative to hysterectomy in high-risk women with atypical endometrial hyperplasia. J. Minim. Invasive Gynecol..

[69] Wells M., Sturdee D.W., Barlow D.H., Ulrich L.G., O’Brien K., Campbell M.J., Vessey M., Bragg A. (2002). Effect on endometrium of long term treatment with continuous combined estrogen-progestogen replacement therapy: Follow up study. BMJ.

[70] Sturdee D.W., Ulrich L.G., Barlow D.H., Wells M., Campbell M.J., Vessey M.P., Nielsen B., Anderson M.C., Bragg A.J. (2000). The endometrial response to sequential and continuous combined estrogen-progestogen replacement therapy. BJOG.

[71] Fisher B., Costantino J.P., Wickerham D.L., Cecchini R.S., Cronin W.M., Robidoux A., Bevers T.B., Kavanah M.T., Atkins J.N., Margolese R.G. (2005). Tamoxifen for the prevention of breast cancer: Current status of the National Surgical Adjuvant Breast and Bowel Project P-1 study. J. Natl. Cancer Inst..

[72] Chin J., Konje J.C., Hickey M. (2009). Levonorgestrel intrauterine system for endometrial protection in women with breast cancer on adjuvant tamoxifen. Cochrane Database Syst. Rev..

[73] Shi Q., Li J., Li M., Wu J., Yao Q., Xing A. (2014). The role of levonorgestrel-releasing intrauterine system for endometrial protection in women with breast cancer taking tamoxifen. Eur. J. Gynaecol. Oncol..

[74] Wong A.W., Chan S.S., Yeo W., Yu M.Y., Tam W.H. (2013). Prophylactic use of levonorgestrel-releasing intrauterine system in women with breast cancer treated with tamoxifen: A randomized controlled trial. Obstet. Gynecol..

[75] Kelly P., Dobbs S.P., McCluggage W.G. (2007). Endometrial hyperplasia involving endometrial polyps: Report of a series and discussion of the significance in an endometrial biopsy specimen. BJOG.

[76] De Rijk S.R., Steenbergen M.E., Nieboer T.E., Coppus S.F. (2016). Atypical endometrial polyps and concurrent endometrial cancer: A systematic review. Obstet. Gynecol..

[77] Joshua A., Allen K.E., Orsi N.M. (2025). An Overview of Artificial Intelligence in Gynaecological Pathology Diagnostics. Cancers.

[78] Rewcastle E., Gudlaugsson E., Lillesand M., Skaland I., Baak J.P.A., Janssen E.A.M. (2023). Automated Prognostic Assessment of Endometrial Hyperplasia for Progression Risk Evaluation Using Artificial Intelligence. Mod. Pathol..

